# 

**Published:** 1910-12-03

**Authors:** 


### THE HOSPITAL

Telegraphic Address: Ospedale, London.

THE MODERN MEDICAL AND INSTITUTIONAL JOURNAL.

Telephone Number: 2734 Gerrard.

NEW SERIES. No. 199, Vol. VIII. No. 1271, Vol. XLIX.

SATURDAY, DEC. 3RD, 1910.

PRICE THREEPENCE

